# CODC-v1: a quality-controlled and bias-corrected ocean temperature profile database from 1940–2023

**DOI:** 10.1038/s41597-024-03494-8

**Published:** 2024-06-22

**Authors:** Bin Zhang, Lijing Cheng, Zhetao Tan, Viktor Gouretski, Fuchao Li, Yuying Pan, Huifeng Yuan, Huanping Ren, Franco Reseghetti, Jiang Zhu, Fan Wang

**Affiliations:** 1 Institute of Oceanology, Chinese Academy of Sciences, Qingdao, 266071 China; 2 Center for Ocean Mega-Science, Chinese Academy of Sciences, Qingdao, 266071 China; 3 Oceanographic Data Center, Chinese Academy of Sciences, Qingdao, 266071 China; 4 International Center for Climate and Environment Sciences, Institute of Atmospheric Physics, Chinese Academy of Sciences, Beijing, 100029 China; 5 University of Chinese Academy of Sciences, Beijing, 101408 China; 6 Computer Network Information Center, Chinese Academy of Sciences, Beijing, 100039 China; 7 Istituto Nazionale di Geofisica e Vulcanologia (INGV), Bologna, 40127 Italy

**Keywords:** Physical oceanography, Physical oceanography

## Abstract

High-quality ocean *in situ* profile observations are fundamental for ocean and climate research and operational oceanographic applications. Here we describe a new global ocean subsurface temperature profile database named the Chinese Academy of Science (CAS) Oceanography Data Center version 1 (CODC-v1). This database contains over 17 million temperature profiles between 1940–2023 from all available instruments. The major data source is the World Ocean Database (WOD), but CODC-v1 also includes some data from some Chinese institutes which are not available in WOD. The data are quality-controlled (QC-ed) by a new QC system that considers the skewness of local temperature distributions, topographic barriers, and the shift of temperature distributions due to climate change. Biases in Mechanical Bathythermographs (MBTs), eXpendable Bathythermographs (XBTs), and Bottle data (OSD) are all corrected using recently proposed correction schemes, which makes CODC-v1 a bias-corrected dataset. These aspects ensure the data quality of the CODC-v1 database, making it suitable for a wide spectrum of ocean and climate research and applications.

## Background & Summary

The increasing concentration of greenhouse gases in the atmosphere creates a net positive radiative forcing in the climate system. In response, the Earth’s surface warms, triggering feedback in the climate system^1^. At the same time, heat penetrates into the ocean, resulting in the warming of ocean waters, and increasing ocean heat content (OHC)^2–4^. Ocean warming has consequences, such as strengthening tropical cyclones, fuelling extreme events, and raising the global sea level^5^. Meanwhile, ocean warming and stronger vertical stratification decrease the vertical exchange of oxygen, leading to ocean deoxygenation and threatening marine life^6,7^. Even if society can slow all greenhouse gas emissions and achieve the “net zero” by mid-century as targeted by the United Nations Paris Agreement, there is a lag built into the climate system primarily as a result of the ocean thermal inertia with changes such as deep ocean warming and sea-level rise continuing for at least several hundred years^1,5^.

The influence of oceanic changes on social and economic systems depends on the physical state of the ocean and the local vulnerability to changes, as well as on worldwide efforts to mitigate the impact of future climate change and to adapt to current and forthcoming changes. Thus, our knowledge of climate change, particularly of ocean warming, can be used to support social adaptation and mitigation decisions. High-quality and bias-free *in situ* observations are critical for detecting statistically significant changes and monitoring the variability of the climate. Therefore, maintaining, managing, and improving the global archive of ocean data is crucial for climate actions and operational oceanography applications.

Two groups of data-processing techniques are critical to ensure a high-quality dataset: (1) data quality control (QC), which removes incorrect outliers (e.g., observations that differ significantly and erroneously from the majority of the data in the population), and (2) bias correction, which seeks to eliminate systematic errors in the data.

Many QC-ed datasets/products have been provided in the past decades by several research groups, each usually linked to a specific database, for example, World Ocean Database (WOD)^8^; Global Ocean Data Analysis Project (GLODAP) dataset^9^; Met Office Hadley Centre (EN) dataset^10^; Copernicus *in situ* ocean dataset of temperature and salinity (CORA)^11^; Argo database^12^; Global Temperature and Salinity Profile Programme (GTSPP)^13^, International Quality-controlled Ocean Database initiative (IQuOD)^14,15^. Each QC system has its strengths and limitations^16^. For example, many existing QC systems assume a Gaussian distribution for regional temperature distributions, which neglects the skewness of the natural temperature variations. Also, most QC schemes do not implement a vertical temperature gradient check, which uses information on the profile shape, thus increasing the ability of the QC scheme to identify outliers. Besides, the long-term trend of the local climatological thresholds in a warming climate has not been accounted for, leading to the exclusion (rejection) of data linked to realistic extreme events. The topographic barriers that separate different water masses are often not taken into account in many QC systems. To resolve these issues, ref. ^17^ proposed a new QC system, which has been shown to be superior in identifying outliers and minimizing the wrong flagging of good data. This new AutoQC system, namely the Chinese Academy of Science (CAS) Oceanography Data Center Quality Control system (CODC-QC), is applied in the new database introduced in this paper.

Systematic errors have been identified in the data collected by several instrument types, namely mechanical bathythermographs (MBTs)^18,19^, expendable bathythermographs (XBTs)^20,21^, Nansen bottle casts^22^ and Argo floats^23^. Thus, biases are common to many ocean instruments and impact data accuracy. For example, a spurious decadal variability due to the bias in XBT data was found in the OHC record from 1970–2001, leading to the underestimation in the estimate of long-term ocean warming rate^24^. Community efforts have been made to understand the errors and improve the data quality. New bias correction schemes have been suggested for XBT, MBT, and Nansen Bottle cast data^19,22,25^, and are implemented in the database under consideration. We underline that the often unstable, if not critical, operating conditions at sea contribute to worsening the quality of marine data as well as a large amount of data recorded with instruments having generally poor quality, mainly until the 1990s. Therefore, the right evaluation and estimate of the uncertainties in the measurements of marine parameters are not simple operations. A significant effort has been made by researchers to understand the problem and find a way to improve the data quality.

Benefiting from the collective progress of QC and bias corrections, this study describes a newly organized ocean *in situ* data archive, named the Chinese Academy of Science Oceanography Data Center version 1 (CODC-v1) database, which is QC-ed and bias-corrected to enable wider use of accurate ocean data both for climate research and in operational applications. Additionally, the availability of the CODC-v1 facilitates further inter-comparison between databases. The difference in QC, bias-correction, and other data processing procedures represents a source of uncertainty in OHC estimates as well as in other applications (i.e., reanalysis product generation), which has not been quantified yet^26^.

Table 1 and Table 2 list the data format and introduces variables in the data files of CODC-v1. In addition, we provide access to the software that enables users to tailor data processing and interpolation methods for their specific purposes.

**Table 1:** A description of the metadata information stored in CODC-v1 (in MATLAB files).

Variables	Description
Tprofile_num_all	Total number of profiles
Date	Year, month, day records
Instrument type	The index for temperature instrument type (1 = OSD; 2 = CTD; 3 = MBT; 4 = XBT; 5 = SUR; 6 = APB; 7 = MRB; 8 = Argo; 9 = DRB; 10 = UOR; 11 = GLD; 12 = DBT; 13 = STD; 14 = microBT; −999 = Unknown)
Country	Country name of the profile
Access_num	Accession number in WOD provided by NODC
Latitude	Latitude record of the profile in degree ([−90 °,90°])
Longitude	Longitude record of the profile in degree ([−180 °,180^o^])
GMT_time	Observation time zone (GMT)
Time	observation time since: 1770-01-01 00:00:00
CAS_unique_cast	Profile unique ID provided by CODC-v1
WOD_unique_cast	Profile unique ID provided by NOAA/NCEI (WOD)
WOD_cruise_identifier	Cruise ID provided by NOAA/NCEI
WMO_id	ID provided by the World Meteorological Organization
Launch_height	The launch height above the sea surface level (only for XBT data)
Need_depth_fix_instrument	Index of whether the XBT depth bias correction is needed (only in XBT data)
XBT_depth_eq	The type of equation used for XBT depth bias correction (only in XBT data)
Platform	Platform name
Project	Project name
Real_time	The flag index of whether the Argo data are real-time data or not (real-time; real-time adjusted; delayed-mode)
Temp_instrument	The probe type of the temperature instrument
Recorder	The recording system type and version of the temperature instrument

**Table 2:** A description of the temperature profile data and their flags stored in CODC-v1 (in MATLAB files).

Variables	Description
Depth	Depth levels after CODC-QC and XBT/MBT/Bottle bias correction
Temperature	Temperature measurements after CODC-QC and XBT/MBT/Bottle bias correction
Temp_origin	Original temperature data without preforming any bias corrections
Depth_origin	Original depth data without preforming any quality control and bias corrections
Temp_XBTcor	Corrected temperature records after CH14 XBT bias correction (only for XBT data)
Depth_XBTcor	Corrected depth records after CH14 XBT bias correction (only for XBT data)
Temp_MBTcor	Corrected temperature records after GC20 MBT bias correction (only for MBT data)
Depth_MBTcor	Corrected depth records after GC20 MBT bias correction (only for MBT data)
Temp_BOTcor	Corrected temperature records after GBC22 bottle bias correction (only for bottle data)
Depth_BOTcor	Corrected depth records after GBC22 bottle bias correction (only for bottle data)
Temperature_CODCflag	Temperature QC flag provided by CODC-QC at each observed level (0 is good data; 1 indicates at least one QC checks failed)
Temperature_CODCflag_checks	14 QC flags corresponding to each distinct check of CODC-QC at each observed level (0 indicate passing an individual check; 1 means a failed individual check)
Temperature_WODflag	The temperature QC flag provided by WOD18 (NOAA-NCEI) at each observed level, for comparison with CODC-QC
Depth_WODflag	The depth QC flag provided by WOD18 (NOAA-NCEI) at each observed level, for comparison with CODC-QC

## Methods

### Workflow overview

#### Data sources

The primary data are obtained from *in situ* measurements available through the World Ocean Database (WOD) downloaded in March 2023^8^. The instruments include XBT^27^, Argo^12^, Conductivity/Temperature/Depth (CTD), MBT, bottle, moored and buoy (MRB), Drifting buoy (DRB), glider (GLD), Autonomous Pinniped data (APB)^28^ and others. Both Delayed-mode and Real-time mode Argo data are assembled in CODC-v1. Since this database will be updated regularly at least on an annual basis as in ref. ^29^, newly available Delayed-Mode Argo data from the Argo Data Centre will be added and replace the Real-time data during the update process (every 1~4 months). Besides, some unique data owned by several institutes and not yet available through the WOD and GTSPP are included in CODC-v1. These unique data include some publicly available data^30–36^ and previously archived data by CODC. There is a total of 17,657,649 profiles from January 1940 to December 2023 in CODC-v1 (with 22,784 from Chinese institutes), which are all publicly accessible.

#### Instrumentation types and spatial and temporal data coverage

**Fig. 1:**
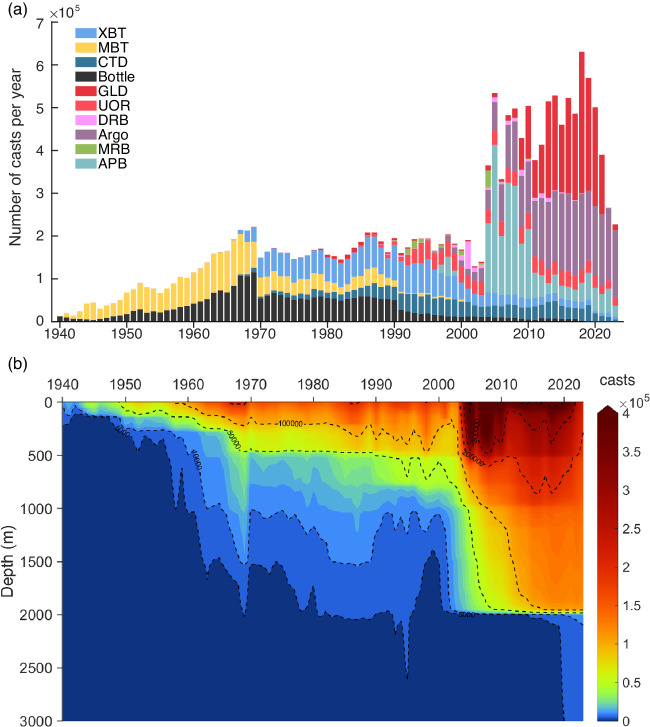
Yearly number of temperature profiles in CODC-v1. (**a**) Annual number of temperature profiles collected by different instruments in the CODC-v1. (**b**) Yearly number of observations at different layers. Panel (b) was produced using profiles interpolated to the standard levels.

**Fig. 2:**
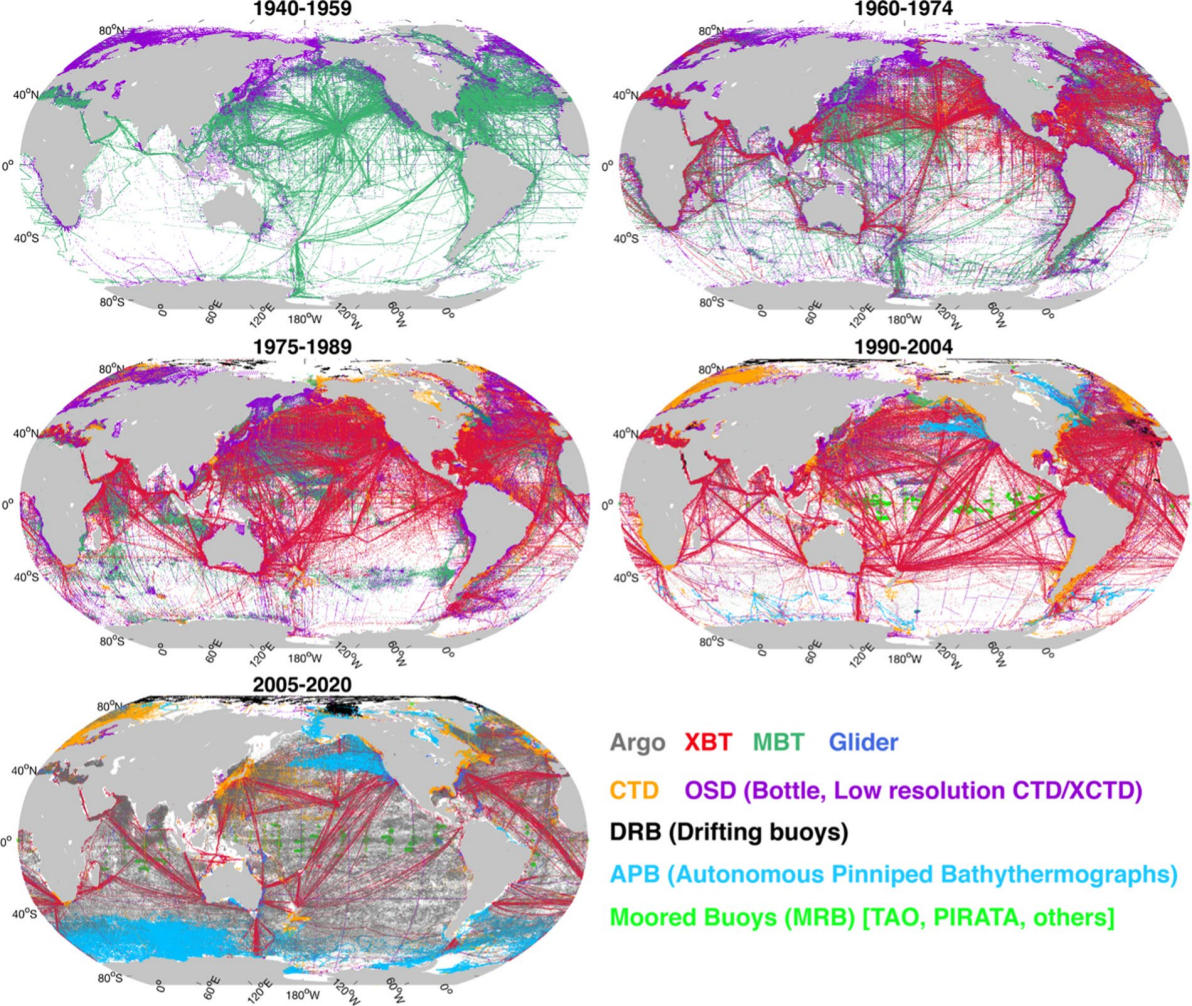
Geographical distribution of profiles from different instruments in CODC-v1 for several decadal periods.

The data distribution over time for different instruments is illustrated in Fig. 1a,with the spatial distribution of data shown in Figs. 1b and 2a. The Nansen Bottle (used as BOT before, but OSD in this paper), invented at the end of the 19^th^ century, has been used to measure the seawater temperature at depths from 1940 until now. There are ~2.61 million profiles within the OSD instrumentation category (ocean station data, including bottle and low-resolution CTD profiles), which are mainly distributed in the coastal regions of the Northern Hemisphere (Fig. 2).

MBTs (invented around 1930) measured the temperature in the layers down to the depth of ~300 m, and was the main component of the database in the period 1940-1965^19^. They have a much wider geographic coverage than the OSD, extending over the entire Northern Hemisphere, mainly along busy trade lines connecting continents and between countries (Fig. 2).

Since the end of 1965, XBT probes have been widely deployed, first by Navies and soon adopted by oceanographers. They constitute the most abundant component of the database during the period 1966–2001 and still play a critical role today (Figs. 1a, 2)^21,27^. There are different models of XBT probes, with two large families that reach depths of 450 m (shallow) and 750 m (deep), respectively. The shallow version was the first invented and mainly used until 1990 (Fig. 1b), progressively replaced by the deep model which became the most widespread in the database in the period up to the 2000s (Fig. 1b)^37,38^. Due to its affordability and relative ease of use, XBT probes were used extensively in the three decades from the late-1960s to 2001 in the global ocean, with a significant component recorded within the Ship of Opportunity Program (SOOP) activity, which exploits commercial ships. There is a total of approximately 2.35 million XBT profiles in CODC-v1, but spatial sampling gaps are evident, especially in the South and Southeast Pacific.

The CTDs have also been widely used since the 1960s mainly by research vessels, they typically observe ocean temperature from the sea surface down to at least 1000 m (but the maximum depth is flexible) (Fig. 1b). Because of the high accuracy of CTDs, CTD data are always regarded as the “golden standard” of the ocean observations. During the 1990s, increased subsurface measurement coverage of the global ocean by means of high-quality hydrographic sections (CTDs are mainly used) was achieved as part of the World Ocean Circulation Experiment (WOCE)^39,40^.

Since the beginning of this millennium, the Argo array of autonomous floats began to monitor the upper 2000 m of the open ocean (Fig. 1a,b)^41^. After a few years to settle the structure, the Argo programme achieved a homogeneous and isotropic distribution of temperature profiles guaranteed by about 4000 floats operating simultaneously and providing ~12000 profiles per month. The type of sensor installed, completely equivalent to that of the CTDs, guarantees high quality and is also subjected to a continuous qualitative review process. Its advent, integrated with other measuring instruments, has opened a new era of the global ocean observation system allowing for the first time the global coverage of the open ocean (Fig. 2). There are currently approximately 2.70 million Argo profiles in CODC-v1.

The APB profiles were collected by programs that equipped marine animals with temperature and salinity data loggers^42^. There are ~2.04 million APB temperature profiles in the CODC-v1 (Fig. 1a), primarily located in the regions where these animals live, i.e. in the high-latitude coastal regions (Fig. 2)^28^. The overall quality of this data is still under analysis and the presence of potential bias in the data collected by some types of sensors is suspected.

The glider (GLD), an autonomous, unmanned underwater vehicle that requires little or no human assistance during travel, provides a mass of profile observations near coastlines (Fig. 2)^43^. The type of instrumentation installed is identical to that of the CTDs, therefore capable of providing excellent data quality. There are ~2.5 million GLD profiles in total. Although the amount of APB and GLD profiles is comparable to that of Argo profiles, they have a limited geographical coverage (Fig. 2).

#### Data processing

**Fig. 3:**
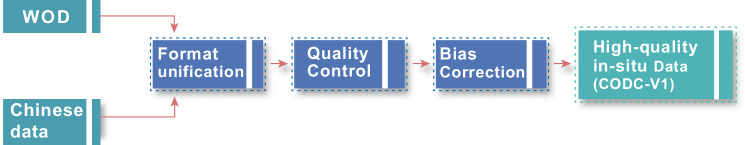
Flowchart illustrating the main workflow, data source, and techniques used in CODC-v1.

**Fig. 4:**
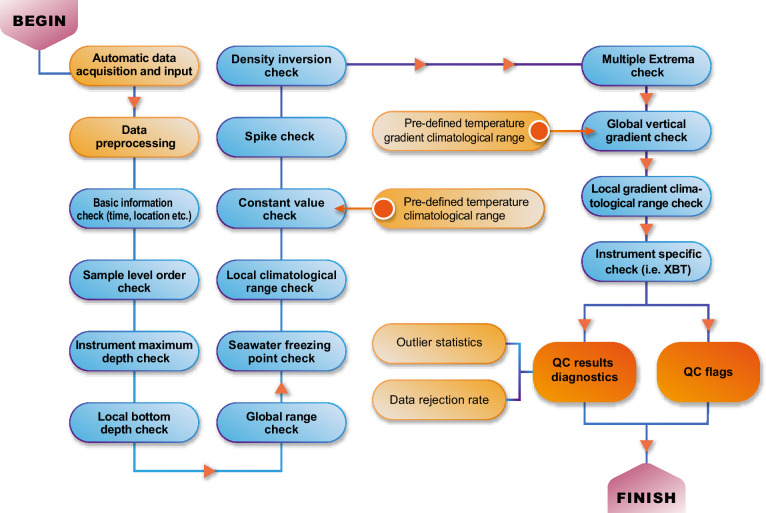
Flowchart illustrating the QC workflow in the CODC-v1 system. The blue icons indicate individual QC modules, and the red icons show the system’s pre-defined thresholds and other data processing procedures. Here, the CODC-QC system^17^ is used, with the source codes available in the Code Available section.

The data are processed following the data processing flow shown in Fig. 3. First, all the data have been unified with an internal data format, facilitating the follow-on data processing. The duplicates are also removed at this step. Because the raw *in situ* data are of heterogeneous quality, a unified QC system is essential to homogenize the data and to ensure the highest possible level of consistent quality across the entire database^15,17,44^. This database has been QC-ed by a newly proposed CODC-QC system^17^ (latest version in November 2023) that includes 14 distinct quality checks, illustrated in Fig. 4. Besides the basic information checks, the system compares each observation with predefined climatological ranges (e.g., global range check, seawater freezing point check, local climatological range check) and also checks of the profile shape (e.g., spike check, constant value check, local vertical gradient range check) and checks related to the specific instrumentation (e.g., XBT instrument check).

The key advantages of this QC system include: (1) accounting for the anisotropic feature of the local temperature distribution (2) accounting for topographic barriers separating different water mass properties; (3) local climatological ranges vary with time to account for ocean warming, reducing the possibility of erroneously excluding true extreme events; (4) all QC modules are optimized with a diagnostic tool^45^ and then they are also evaluated manually; (5) the CODC-QC system has been evaluated using two benchmark datasets, indicating the system skill in removing spurious data and minimizing the percentage of mistakenly flagged good data^17^.

The bias in XBT data was first reported soon after its invention in the early 1960s, however, the global impact of this bias was not fully recognized until 2007^20^. Since then, many studies have been devoted to understanding the error sources and proposing correction schemes^21,27,46^. It is now well established that the XBT bias has two components related to depth and temperature measurements, respectively^47,48^. The depth bias is due to the absence of a pressure sensor and the manufacturer’s equation describing the fall rate of XBT probes does not accurately describe the actual probe motion. The temperature bias can be traced back to the temperature sensor, the acquisition system, and in general to the electrical-electronic part of the XBT system (probes + acquisition unit), etc. Biases in the XBT data were minimized using the correction scheme shown in ref. ^25^ (hereinafter CH14 scheme), which corrects both depth and temperature biases and depends on water temperature, probe type, and time. Further evaluations confirmed that CH14 may better correct local and global biases in XBT data than other schemes^49^, therefore CH14 is currently recommended by the oceanographic community^21,26,27^.

MBT data dominated the global ocean database from 1940 to 1970. The MBT temperature bias depends on the instrument type, data acquisition procedure, and data processing procedure, for instance, temperature and pressure sensor response delay, inaccurate grid viewer calibration, and data reading system errors. A correction scheme has recently been described in ref. ^19^ (named GC20 hereinafter) and is capable of correcting for both depth and temperature biases, showing a dependence on country, depth and time. Therefore, the time-varying bias is corrected separately for country-specific profiles, including the United States, Soviet Union (Russia), Japan, Canada, and Great Britain. Data from all other countries are corrected with globally averaged correction factors (for depth and temperature separately) calculated by all MBT data, which is also time varying (i.e., annual correction).

Bottle data contributes a significant fraction of data before 1990, and the recent analysis quantified bottle data temperature bias to be ~0.05 °C before 1980 on 0–700 m average^22^. A correction scheme was recently proposed in ref. ^22^ (named GCB22 from now on) and applied in the CODC-v1 database in this study. Both the depth and temperature measurements of the bottle data are biased and vary with the calendar year. Temperature bias is constant with depth, while depth bias varies with depth.

## Data Records

QC-ed and Bias-corrected data of CODC-v1^50^ are available from the Chinese Academy of Sciences Ocean Data Repository at 10.12157/IOCAS.20230525.001, and no registration is needed to download the dataset. The product includes one “.mat” (MATLAB format) and “.nc” (NetCDF format) file per month, which collects all data in a particular month. The data in the two formats are identical, so Table 1 and Table 2 only describe the variables in the MATLAB format. Besides, because the main data source is WOD, we have kept all WOD metadata information, for example, the WOD QC flags and WOD data ID. This consistency is essential for the future comparison of CODC-v1 with WOD, for instance, investigating the impacts of different QC and bias-correction on OHC estimate, ocean reanalysis product generation, and ocean prediction. Additionally, we also note that the CODC database is different from the Institute of Atmospheric Physics (IAP) datasets, which are referred to as gridded datasets after spatial interpolation but with the CODC *in situ* profile database as an input.

For the entire database, 7.18% of measurements (e.g., 183,414,409 data points) are rejected by CODC-QC, in which the CTD and Argo data exhibit the lowest rejection rate. In comparison, the XBT (13.84%), Glider (7.48%), and Nansen Bottle (6.55%) are characterized by the top-3 highest rejection rates. In the near-surface layer (0–50 m), 7.41% of data were rejected, with MRB and DRB data exhibiting the highest percentage. Within 50–400 m, 5.56% of data were rejected, dominated by MBT data; while within 400–1000 m, 8.14% of data were rejected, dominated by XBT data; 1000–2000 m, 3.50% of data were rejected, dominated by APB data; below 2000 m, 4.85% of data were rejected, dominated by Argo data. For Chinese data, 6.17% of measurements are rejected by CODC-QC. This rejection shows a homogenous change with depth in the upper 500 m, primarily ranging from 2.0% to 2.5%. However, it increases with depth from 500 m to 1,000 m.

## Technical Validation

**Fig. 5:**
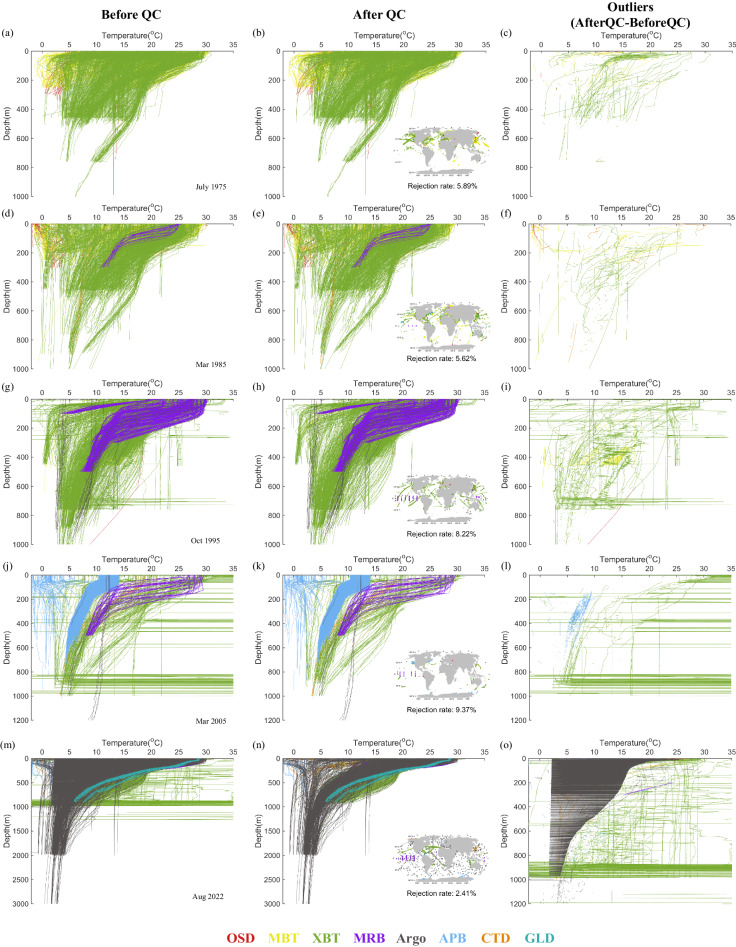
Examples of 3,000 randomly selected temperature profiles before (left panel) QC, after QC (middle panel), and data outliers (right panel). The examples are chosen arbitrarily from several years: (**a**–**c**) July 1975, (**d**–**f**) March 1985, (**g**–**i**) October 1995, (**j**–**l**) March 2005, (**m**–**o**) August 2022. The location of the selected profiles is shown in the insets of the middle panels. Different colors denote different instrument types. The excess of profiles in plots **a**-**b**) **d**-**e**) and **g**-**h**) ending around 450 and 750 m of depth is due to the XBT dominant component (respectively for the shallow and deep models).

The QC system has been thoroughly validated in ref. ^17^, where the detailed description can be found. Figure 5 provides several representative examples showing temperature profiles before and after the CODC-QC system. We randomly selected 3,000 temperature profiles collected by several different instruments in (a-c) July 1975, (d-f) March 1985, (g-i) October 1995, (j-l) March 2005, (m-o) August 2022. These examples illustrate the existence of many erroneous data (e.g., spikes, extreme values, constant values) from different instruments, which have been identified and flagged in CODC-v1. Specifically, some profiles looked suspicious (e.g. the near vertically constant profile around 13 °C in Fig. 5b,e, k, and n) but still passed the CODC-v1 check. After a manual investigation, these profiles are physically plausible as they are from the Mediterranean Sea, characterised by a homogeneous thermal structure with depth. This is an example of the validity of the QC system. Within the 14 modules, the local climatological range check and the local climatological gradient range check are the most effective in detecting outliers.

**Fig. 6:**
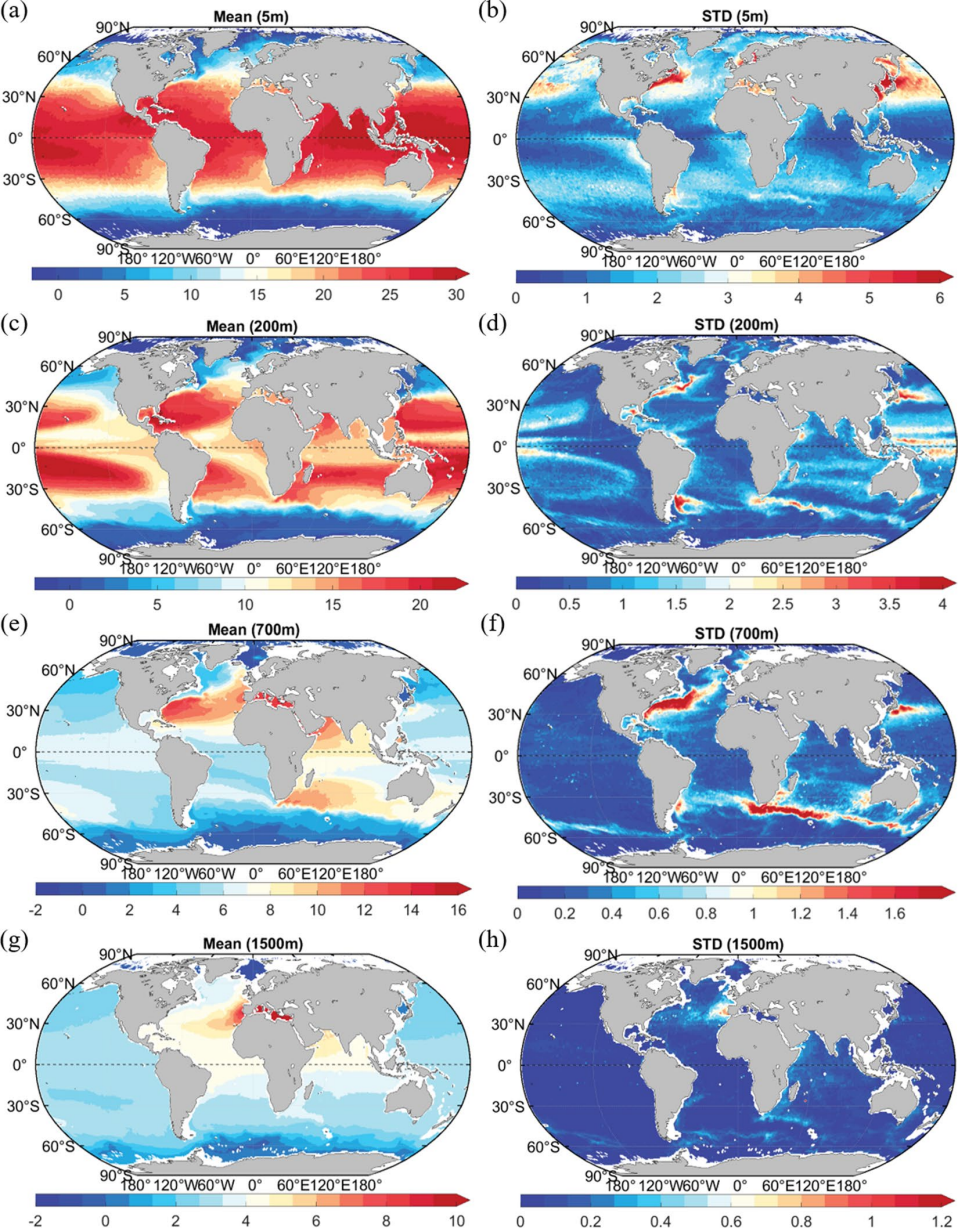
Temperature average and standard deviation on 1° by 1° grid using all CODC-v1 QC-ed and bias-corrected data at different layers: (**a,b**) 5 m, (**c,d**) 200 m, (**e,f**) 700 m, and (**g,h**) 1500 m. The scales are different in different panels. The unit is °C.

Additional validation of the QC-ed data can be made through inspection of gridded averages and standard deviations, which represent the climatological state of the ocean temperature. An effective QC system should be able to delete spikes or any unphysical temperature distributions (Fig. 6 versus Fig. S1). Figure 6 shows the temperature gridded average and standard deviation at several selected layers (5 m, 200 m, 700 m, and 1500 m). The respective spatial patterns appear well-defined and physically meaningful without significant spurious signals. For example, the warm pool (temperature > 28 °C) in the Western Pacific, North Atlantic, and Eastern Indian Ocean, as well as cold polar regions, are all well-defined (Fig. 6a). The warmer waters in the subtropical regions can be seen in the subsurface level (e.g., 200 m in Fig. 6c) associated with a deeper mixed layer and thermocline in these regions. Higher variability occurs in the energetic regions of the Gulf Stream, Kuroshio, and Antarctic Circumpolar Current regions all the way down to at least 700 m level (Fig. 6b,d, f). The deep-reaching Agulhas current is also visible in Fig. 6f down to 700 m. The gridded fields show vertically homogeneous and high temperatures in the Mediterranean Sea. The Mediterranean outflow impacts the Atlantic Ocean, causing higher temperatures and increased temperature variability in the middle Atlantic Ocean at 1500 m (Fig. 6g,h). These physically meaningful patterns suggest the success of the QC system in removing outliers. Instead, for the data without QC (Fig. S1), there appears to be spurious increases in temperature standard deviation in some isolated grid boxes and along some cruise lines, indicating the impact of erroneous data.

**Fig. 7:**
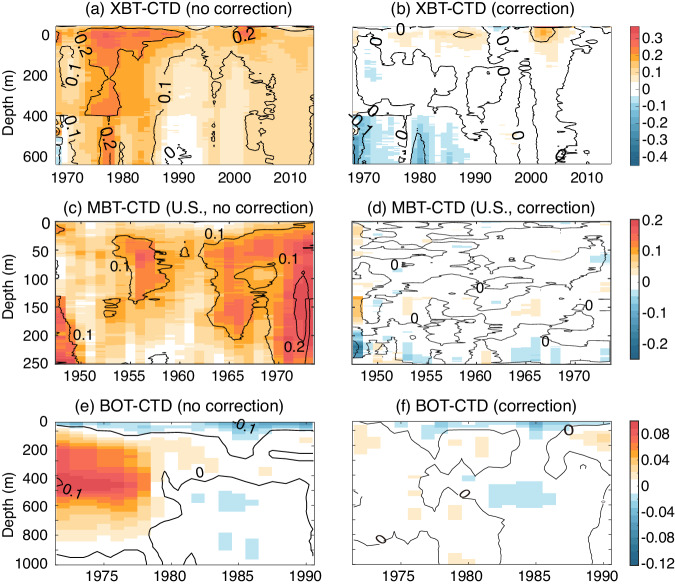
Temperature offset of XBT (**a,b**) and MBT data (**c,d**) (both from the United States of America); Bottle data temperature offset (**e,f**) relative to co-located CTD data. Offsets are shown for the data before (left side) and after (right side) bias corrections.

We compared the original temperature profiles with co-located CTD profiles to assess the impact of bias corrections for XBT, MBT, and OSD temperature profiles (Fig. 7). Based on previous studies^19,22,25,49^, we selected the collocation bubble size of 150 km and the maximum time difference between the observations of ~45 days. Previous studies suggested bias corrections were not sensitive to the slight modification of this choice of time and spatial distance^19,22^. The XBT bias is as large as 0.1~0.2 °C before 1980 on the global 0–700 m average and reduces to less than 0.05 °C after 1990. For the MBT data from the United States of America (which is the major subset of the global MBT archive), the bias is as large as 0.2 °C during the 1970s on the global 0–250 m average, and 0.05 ~ 0.1 °C from the 1940s to the late 1960s. The application of bias corrections can effectively reduce the original bias as demonstrated by Fig. 7c,d. Similar results are found for bottle correction. For OSD data, a positive bias is characteristic during 1970–1980 (~0.05 °C), being larger from the 200 m to 600 m layer than in the other layers. After 1980, the bias diminishes, probably due to the improved data recording techniques. The original time and depth-varying biases can be substantially reduced after applying corrections implemented by GBC22 (Fig. 7e,f). The above results illustrate the impact of bias corrections, substantially reducing the original biases for several instrument types and respectively increasing the homogeneity of the entire database.

**Fig. 8:**
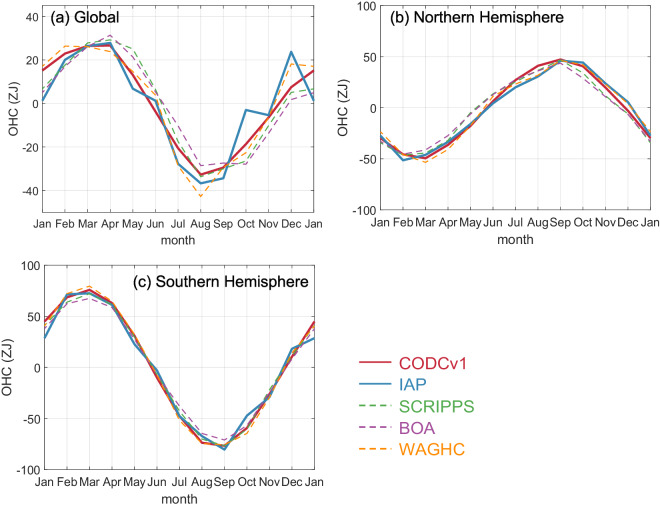
Annual cycle of OHC of upper 2000 m for the global ocean (**a**), the Northern Hemisphere (**b**), and the Southern Hemisphere (**c**) based on the new (red) and old (blue) QC-ed CODC-v1 data. Annual cycles based on three other gridded datasets (SCRIPPS, BOA, and WAGHC) are shown for comparison.

An additional evaluation has been made for the annual cycle of OHC for the upper 2000 m to check if the new QC system results in a better description of the OHC annual cycle. Figure 8 shows the global and hemispheric climatological annual cycle (for the period 2005–2020) of 0–2000 m OHC as calculated on the basis of the CODC-v1 database. For the calculation of the annual OHC cycle, all CODC-v1 data from 2005 to 2020 are averaged together to construct a monthly climatology using the Institute of Atmospheric Physics (IAP) mapping approach^51^. The OHC annual cycle was also calculated based on several available gridded climatologies, including the previous version of the IAP^51^, WAGHC product (WOCE/Argo Global Hydrographic Climatology)^52^ from the University of Hamburg, and two Argo-only gridded products—the Scripps Institution of Oceanography (SCRIPPS)^53^ and the Barnes objective analysis Argo (BOA)^54^.

The global OHC appears to reach its maximum from March to April being at a minimum from August to September (Fig. 8a). The two hemispheres show opposite OHC annual variation, with peak values in March for the Southern Hemisphere and in September for the Northern Hemisphere (Fig. 8b,c). These variations are associated with the Earth’s orbital movement and with the uneven distribution of land and sea^55^. The opposite changes in the two hemispheres are largely cancelled with each other when deriving the global OHC so that the data errors will stand out. This is a reason why the global-scale quantity can be a good evaluation of the data quality. Compared with the previous IAP version where WOD-QC flags were used for QC, a notable difference of the new CODC-v1 OHC annual cycle is its smoothness, compared to several spurious unphysical month-to-month variations such as the big OHC increase from November to December. The new CODC-v1 OHC annual cycle is in better agreement with the other products, especially with the two Argo-only products, which are based on high-quality Argo observations (Fig. 8). We thus conclude that the new CODC-v1 data has the advantage of better-representing ocean temperature variability due to the reduction of the impact of random and systematic errors. An additional investigation (Fig. S2) reveals that the remaining differences between CODC-v1 and other products are mainly sourced from the eddy-rich regions, including Kuroshio, Gulf Stream, and the Southern Ocean. Further work is needed to fully understand such differences.

In summary, the evaluation results presented here and in earlier studies confirm the high data quality of the new CODC-v1 database achieved due to the application of the new QC system and bias correction schemes. We note that the original temperature profiles are also stored in the CODC-v1 database so that the users can trace back all alterations introduced into original observations.

## Usage Notes

The CODC-v1 database is available from https://english.casodc.com/data/metadata-special-detail?id=1614882932386746369 and 10.12157/IOCAS.20230525.001, https://msdc.qdio.ac.cn, and http://www.ocean.iap.ac.cn/ (under the label of Data Service – *In situ* observations). Argo data were collected and made freely available by the International Argo Program and the national programs that contribute to it. (https://argo.ucsd.edu, https://www.ocean-ops.org). The Argo Program is part of the Global Ocean Observing System.

### Supplementary information

Supplementary Material

## Data Availability

The CODC-QC is freely available from GitHub (https://github.com/zqtzt/CODCQC) as an Open-Source Python package under the Apache-2.0 License. The XBT, MBT, and Nansen Bottle bias correction schemes are freely available from the IAP ocean website (http://www.ocean.iap.ac.cn) under the ‘Data service - New techniques’ label.
