# Supplementary material for: CODC-v1: a quality-controlled and bias-corrected ocean temperature profile database from 1940–2023
Source: Sci Data. 2024 Jun 22;11:666. doi: 10.1038/s41597-024-03494-8 (PMC11193783; doi:10.1038/s41597-024-03494-8)
Supplement: Supplementary file 1 — Supplementary Material [file 41597_2024_3494_MOESM1_ESM.pdf]

Supplementary Material for

CODC-v1: a quality-controlled and bias-corrected ocean temperature profile dataset from 1940-2023

Bin Zhang[1,3,7], Lijing Cheng[2,3,5,7*], Zhetao Tan[2,3,5], Viktor Gouretski[2,3], Fuchao Li[1,3,7], Yuying Pan[2,3,5], Huifeng Yuan[4,5], Huanping Ren[1,3,7], Franco Reseghetti[6], Jiang Zhu[2,3,5], Fan Wang[1,3,7*]

1. Institute of Oceanology, Chinese Academy of Sciences, Qingdao, China, 266071.

2. International Center for Climate and Environment Sciences, Institute of Atmospheric Physics, Chinese Academy of Sciences, Beijing, China, 100029.

3. Center for Ocean Mega-Science, Chinese Academy of Sciences, Qingdao, China, 266071.

4. Computer Network Information Center, Chinese Academy of Sciences, Beijing, 100039.

5. University of Chinese Academy of Sciences, Beijing, China, 101408.

6. Istituto Nazionale di Geofisica e Vulcanologia (INGV), Bologna, Italy, 40127.

7.Oceanographic Data Center, Chinese Academy of Sciences, Qingdao, China, 266071.

* Correspondent authors: Lijing Cheng: chenglij@mail.iap.ac.cn and Fan Wang: fwang@qdio.ac.cn

Submitted to *Scientific Data*

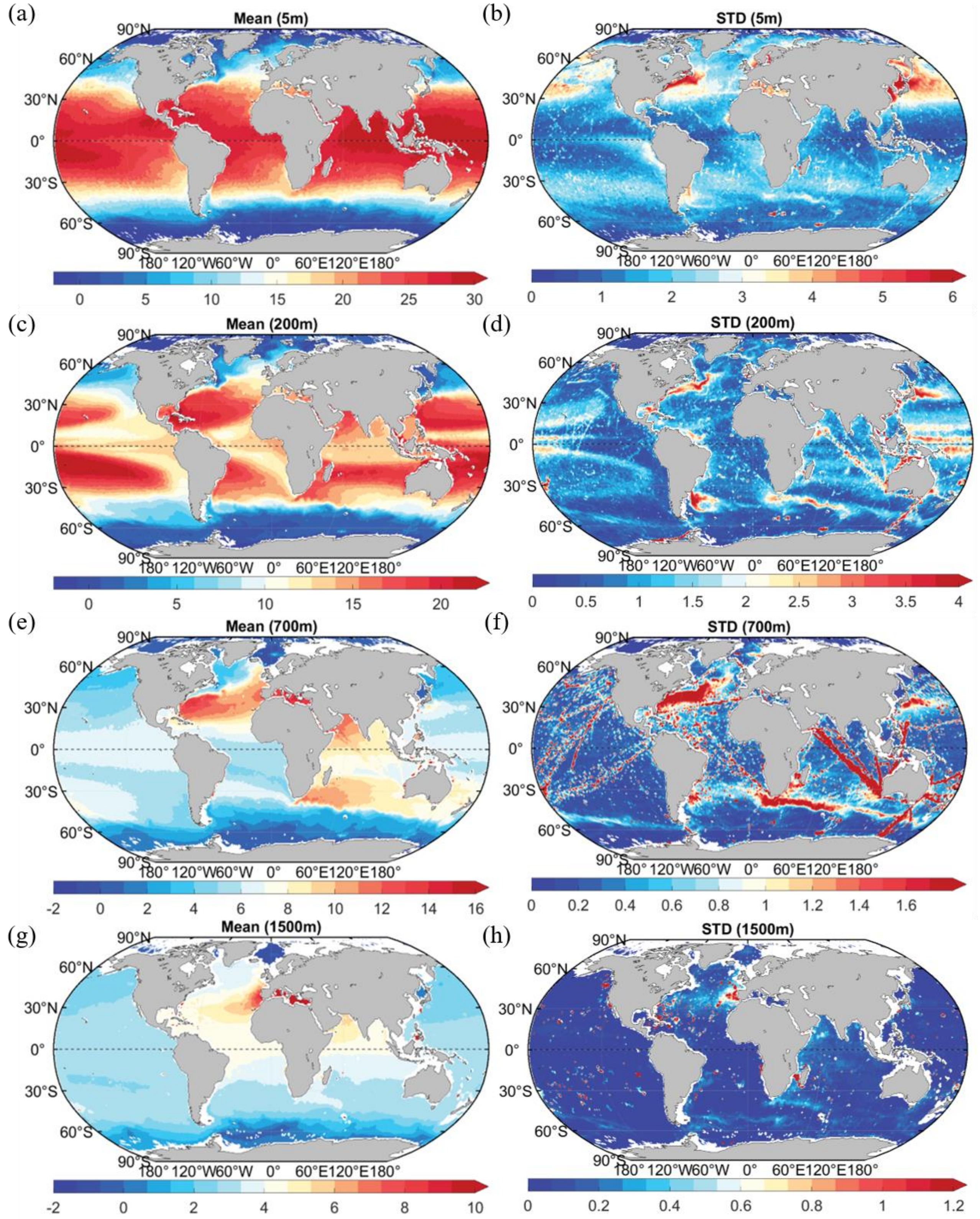

Figure S1. Temperature average and standard deviation on 1-degree box using all CODCv1 bias-corrected data but without performing CODC-QC: (a-b) 5 m, (c-d) 200 m, (e-f) 700 m, and (g-h) 1500 m. The scales are different in different panels. The unit is °C. Here, the gross error data (greater than 35 °C and less than -2 °C) and missing values (sometimes there are NaN, but sometimes there are 999 or others) are removed to avoid absurd results.

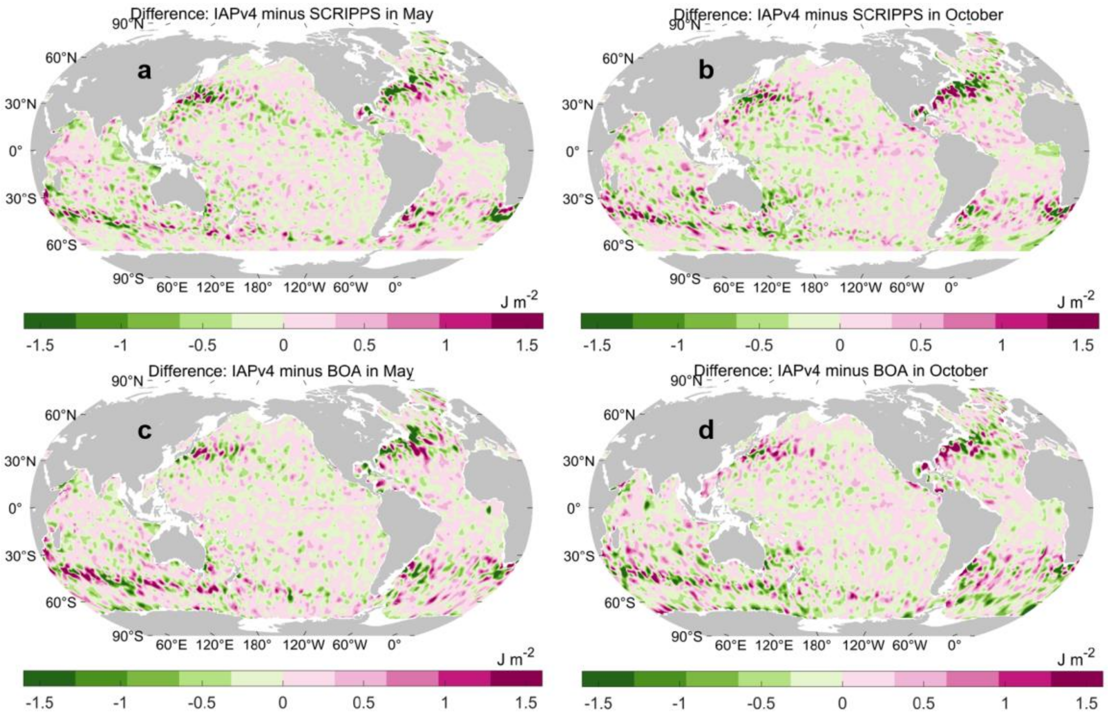

Figure S2. The differences of OHC0-2000m between (a) IAPv4 and SCRIPPS in May, (b) IAPv4 and SCRIPPS in October, (c) IAPv4 and BOA in May, (d) IAPv4 and BOA in October.
